# Comparison of Doppler ultrasound indices of uterine artery and sub endometrial blood supply in frozen embryo transfer with and without repeated implantation failure: A cross-sectional study

**DOI:** 10.18502/ijrm.v21i11.14657

**Published:** 2023-12-19

**Authors:** Fatemeh Bayati, Maryam Eftekhar, Nahid Homayoon, Haniyeh Fatehi

**Affiliations:** Research and Clinical Center for Infertility, Yazd Reproductive Sciences Institute, Shahid Sadoughi University of Medical Sciences, Yazd, Iran.

**Keywords:** Doppler ultrasound, Frozen embryo transfer, Repeated implantation failure.

## Abstract

**Background: **Uterine blood supply has been identified as a potential factor in implantation failure. **Objective:** This study aimed to investigate Doppler indices in the uterine artery, including vascular flow and resistance, as well as the amount of sub-endometrial blood supply in women with a history of repeated implantation failure (RIF) compared to the non-RIF group. **Materials and Methods:** This cross-sectional study was conducted with 139 women candidates for frozen embryo transfer in Yazd Reproductive Sciences Institute, Yazd, Iran from February to July 2023. Group A (n = 68) included women with a history of more than 2 RIF, and group B (n = 71) included women candidates for implantation for the first time without RIF. Doppler ultrasound indices of uterine artery and sub-endometrium, including sub-endometrial flow, uterine artery flow, uterine artery resistance, and peak systolic velocity, were recorded. **Results:** No significant differences were observed in uterine artery Doppler pulsatility index and peak systolic velocity between groups, but the uterine artery resistance index was significantly higher in the A group (p < 0.001). A significant difference was observed in the perfusion area between groups. 60/68 women in the group A had endometrial perfusion in areas 2 and 3 (p < 0.001). **Conclusion:** Our study revealed that women with RIF exhibited higher resistance index in sub-endometrial arteries compared to the non-RIF group.

## 1. Introduction

Implantation failure occurs when a good-quality embryo fails to result in a successful pregnancy in the uterine cavity. There are disagreeing ideas about the exact description of recurrent implantation failure (RIF). According to the latest definition from the European Society of Human Reproduction and Embryology good practice recommendations for RIF, it can be introduced as at least 2 euploid embryo transfers (ETs) had not led to the pregnancy (1).

The process of embryo implantation is complex and relies on the coordination between a high-quality embryo and a receptive endometrium (2-5). A set of characteristics of the endometrium that increases its acceptance for successful implantation and intrauterine pregnancy is still not fully understood. Of course, it seems that the release of female hormones, the most important of which are estrogen and progesterone, is one of the reasons for the change in endometrial acceptance (4, 6, 7). Uterine blood supply has been identified as a potential factor in implantation failure.

Differences in the amount of blood supply to the endometrium between fertile and infertile women have been observed, suggesting that disrupted uterine blood circulation may contribute to implantation failure in infertile women undergoing in vitro fertilization (IVF) (4). Studies investigating the role of Doppler ultrasound indicators have examined the relationship between uterine/endometrial perfusion and implantation. Decreased blood supply in the endometrium and abnormal speed of blood flow in the uterine artery have been identified as important factors in unexplained infertility (3).

The speed of blood flow in the uterine artery is known to increase between the primary follicular phase and the time of implantation. Uterine perfusion plays a crucial role in endometrial acceptance, and disruption of uterine blood supply that can impact implantation failure in infertile women undergoing IVF. The amount of blood circulation in reproductive tissues, including the endometrium and the maturation of follicles, is essential for successful pregnancy (8). Studies have found that relatively low resistance index (RI) and pulsatility index (PI) in the endometrial vessels during the ET cycle are associated with successful pregnancy. Advanced ultrasound techniques, which are non-invasive and allow for the evaluation of the endometrium, have gained attention in this field due to their advantages of real-time monitoring, predictability, and repeatability (9). Although using Doppler ultrasound to assess uterine artery flow and resistance, as well as sub-endometrial blood supply, as a prognostic tool for endometrial acceptance in assisted reproductive cycles is not yet firmly established, it has shown promise in limited studies.

This study aimed to investigate Doppler indices in the uterine artery, including vascular flow and resistance, as well as the amount of sub-endometrial blood supply, in women with a history of repeated implantation failure compared to non-RIF group.

## 2. Materials and Methods

This cross-sectional study was conducted on 139 women who were candidates for frozen embryo transfer (FET) and referred to Yazd Reproductive Sciences Institute, Yazd, Iran from February to July 2023.

Group A (n = 68) included women with a history of more than 2 RIF, and group B (n = 71) included women candidates for implantation for the first time without RIF or a history of successful assisted reproductive technology (ART) leading to clinical pregnancy.

Considering the confidence level of 95%, power of 80%, standard deviation of 0.2 for RI, and the average RI of 0.8 in control and 0.7 for case group using the following formula, and based on similar studies (3, 10), the minimum required sample size was estimated to be 65 in each group. 


$$n=\frac{{\left(Z_{1-\frac{\alpha }{2}}+Z_{1-\beta }\right)}^{2}\times (p_{1}\left(1-p_{1}\right)+p_{2}\left(1-p_{2}\right))}{{\left(p_{2}-p_{1}\right)}^{2}}$$

Women aged between 18 and 40 yr who were candidates for FET with and without RIF and had at least one good quality frozen embryo were included in the study. Women with related autoimmune and metabolic disease (lupus, anti-phospholipid, etc.), any malformation or Asherman, severe male factor infertility, Peri implantation Genetic Test candidates, surrogacy, and donated embryos were excluded.

Each participant had to undergo a standard protocol for the FET cycle. Women with frozen embryos from the previous cycle or on the second day of menstruation in the current cycle underwent a vaginal ultrasound examination conducted by an obstetrics and gynecology specialist. If the ultrasound was normal, they underwent endometrial preparation with 6 mg of estradiol valerate (Abureyhan Pharmaceutical Co., Iran) from the second day of menstruation. Ultrasound was performed on days 12 and 13 of the cycle, and when the thickness of the endometrium reached more than 7 mm, vaginal progesterone 400 mg every 12 hr, along with 50 mg intramuscular progesterone, was administered for 2 days. After the endometrium reached a thickness of more than 7 mm, during the return cycles from freezing, a Doppler examination of the uterine artery and sub-endometrium was performed.

Demographic information, including age, duration of infertility, body mass index, cause of infertility, and laboratory information (number of eggs, and embryo grade), were extracted and recorded in a checklist. Doppler ultrasound indices of uterine artery and sub endometrium of both groups, including sub-endometrial flow, uterine artery flow, uterine artery resistance, and peak systolic velocity (PSV), were also recorded by a single gynecologist using a 7.5 MHZ vaginal probe of a Philips affinity70 machine. The inosation angle was kept below 30 degrees, and RI (RI: PSV-end diastolic velocity/PSV) and PI (PI: PSV-end diastolic velocity/mean velocity) were measured in a sagittal view of the uterus. The vascular flow of the uterine artery was checked bilaterally at the level of the internal hole and lateral to the cervix in the ascending branch of the uterine artery. Endometrial blood supply measured by power Doppler and according to the presence of spiral blood vessels in 3 areas of the endometrium determined: zone 1, endomyometrial junction; zone 2, 5 mm of sub-endometrial area; and zone 3, hyper-echo endometrial edge.

### Ethical considerations

This study was approved by Yazd Reproductive Sciences Institute, Yazd, Iran Ethics Committee (Code: IR.SSU.RSI.REC.1401.019). All procedure was based on the Declaration of Helsinki and Institute ethical statements. Written informed consent was obtained from all participants.

### Statistical analysis

Data were analyzed using social science statistics references, version 22.0 (SPSS Inc., Chicago, IL). Data analysis used descriptive (frequency distribution table, mean ± SD, and median) and analytical statistics (Independent *t *test, Mann-Whitney U, and Chi-square test). A p-value < 0.05 was considered significant.

## 3. Results

Statistical analysis of 139 women candidates for FET in the cleavage stage was performed in this study. The mean age of women in group A was 34.41 ± 3.75 and 31.66 ± 4.65 in group B. Demographic and cycle characteristics, including body mass index, ET, duration of infertility, and cause of infertility in both groups, were compared (Table I). No significant differences were observed between groups in uterine artery Doppler PI and PSV, but uterine artery RI was significantly higher in the A group (p < 0.001) (Table II). A significant difference was observed in the perfusion area between groups. 66/71 women in group B had endometrial perfusion in areas 2 and 3 and 60/68 women in the group A were in areas 2 and 3 (p < 0.001) (Table III).

**Table 1:** Demographic characteristics of participants


**Variables**		**Group A (n = 68)**	**Group B (n = 71)**	**P-value**
Age (yr) ${\text{*}}^{\text{a}}$		34.41 ± 3.75	31.66 ± 4.65	0.08
BMI (kg/m^2^) ${\text{*}}^{\text{a}}$		25.85 ± 4.13	26.69 ± 5.24	0.29
Endometrial thickness (mm) ${\text{*}}^{\text{b}}$		10.01 ± 1.38	9.43 ± 1.58	0.001
Duration of infertility ${\text{(yr)}}^{\text{*b}}$		7.51 ± 4.57	7.35 ± 4.02	0.94
Cause of infertility**				
	**PCOs**	19 (27.9)	20 (28.2)	
	**Unexplained**	24 (35.3)	19 (26.8)	
	**Mix**	9 (13.3)	23 (32.4)	
	**Tubal**	2 (2.9)	3 (4.2)	
	**Diminished ovarian reserve**	14 (20.6)	4 (5.6)	
	**Ovarian endometriosis**	0	2 (2.8)	0.01
*Data presented as Mean ± SD. a: Independent *t* test. b: Mann-Whitney. **Data presented as n (%). Chi-square test. BMI: Body mass index, PCOS: Polycystic ovary syndrome				

**Table 2:** Comparison of uterine artery Doppler between groups


**Uterine artery Doppler indices**	**Group A (n = 68)**	**Group B (n = 71)**	**P-value**
** ${\text{RI}}^{\text{a}}$ **	1.00 ± 0.29 MD: 0.99/IQR: 0.24	0.83 ± 0.24 MD: 0.84/IQR: 0.30	0.001
** ${\text{PI}}^{\text{a}}$ **	1.99 ± 0.64 MD: 2.02/IQR: 0.95	1.93 ± 0.81 MD: 1.8/IQR: 1.17	0.61
** ${\text{PSV}}^{\text{b}}$ **	16.19 ± 2.48 MD: 2.94/IQR: 0.30	16.32 ± 2.94 MD: 2.48/IQR: 0.35	0.78
Data are presented as Mean ± SD. a: Mann-Whitney test. b: Independent *t* test. RI: Resistance index, PI: Pulsatility index, PSV: Peak systolic velocity, MD: Median, IQR: Interquartile range			

**Table 3:** Comparison of endometrial zone perfusion between groups


**Endometrial grade**	**Group A (n = 68)**	**Group B (n = 71)**	**P-value**
**Zone 1 (n = 13)**	8 (11.8)	5 (7.0)	
**Zone 2 (n = 55)**	36 (52.9)	19 (26.8)	
**Zone 3 (n = 71)**	24 (35.3)	47 (66.2)	0.001
Data presented as n (%). Chi-square test			

## 4. Discussion

This study examined Doppler indices in the uterine artery and sub-endometrial blood supply. The study revealed that the uterine artery RI exhibited a significantly higher value in the group with RIF compared to the B group. However, no significant differences were observed between groups in terms of PI and PSV.

The role of blood flow dynamics in reproductive tissues is crucial for successful endometrial development, implantation, and follicular maturation. Any disturbances in blood supply, such as an increase in uterine artery resistance, can adversely affect endometrial receptivity and conduce to RIF (10-12). In our previous study, we compared the effect of sub-endometrial blood flow and uterine artery Doppler indices in FET, and a significant difference was observed in sub-endometrial perfusion between pregnant and non-pregnant women, in this way, it was more in pregnant women. Uterine PI was also significantly lower in the pregnant group, so we concluded that measuring doppler indices and endometrial blood supply can contribute to the pregnancy rate (2).

In contrast to this study, one study stated that the examination of Doppler ultrasound indices performed with transvaginal ultrasound does not have a determining factor in the result of transfer in frozen-thawed embryos (13).

Our study shows that in women with RIF, a higher resistance in the uterine artery than in women in the control group was seen on the day of the start of progesterone in the return cycle from freezing, and the RI was higher in the RIF group.

These results are similar to the result of a study that the PI in the arcuate artery was not meaningfully different among the groups, but the RI, PI in the sub-endometrial blood flow and RI in the arcuate artery were significantly higher in the RIF group compared to the control group (7). A study was conducted to examine the correlation between the thickness of the endometrium and pregnancy outcomes in various age groups of individuals who underwent ART. The study found that as the endometrial thickness increased from 8-11 mm, the pregnancy rate also increased. However, when the thickness increased from 11-14 mm, the pregnancy rate decreased, and beyond 14 mm, the pregnancy rate was close to zero (14).

On the other hand, several studies reported in 3-dimensional power doppler showed a significant difference in the volume of the endometrium was observed between pregnant and non-pregnant women. Still, no difference was observed in the vascular parameters in the endometrium and sub-endometrium (15). Several factors can play a role in endometrial acceptance and implantation. Endometrial blood supply is one of them. In previous studies, the connection between endometrial thickness and endometrial volume in the success of the ART cycle has been examined. Still, to accurately determine the effect of these factors, researchers scored them by 4 factors (endometrial thickness, endometrial volume, echogenicity, and endometrial central echogenic line) and, peristalsis and blood flow of the endometrium also hold. Based on the above division and the score obtained from the scoring, it was determined that the higher the score, the higher the probability of pregnancy. Regarding the effect of blood flow in the endometrium and endometrial peristalsis, it was found that the more the blood flow, the less or even zero peristalsis, and higher the chance of pregnancy. Therefore, a single ultrasound marker may not completely reveal the receptivity of the endometrium and the endometrial receptivity connected to the thickness, volume, echo, peristalsis, and blood flow of the endometrium (16). Our findings were consistent with prior studies that noted an elevated RI in the uterine artery and sub-endometrial blood flow among women who underwent multiple ETs in IVF compared to a control group with regular reproductive function. Our findings were consistent with prior studies that noted an elevated RI in the uterine artery and sub-endometrial blood flow among women who underwent multiple ETs in IVF, compared to a control group with regular reproductive function (17). It is important to acknowledge that the conflicting results in the mentioned study may have been influenced by a limited sample size, particularly in the control group. Additionally, the evaluation of Doppler indices during days 8 and 9 of the menstrual cycle in that study could account for the discrepancies compared to the current study (18, 19).

## 5. Conclusion

This study revealed that women with RIF exhibited higher RI in sub-endometrial arteries compared to group B. Other studies have also linked implantation failure in IVF cycles with decreased or absent blood flow in the sub-endometrium. When assessing endometrial receptivity, it is crucial to consider multiple factors, including Doppler indices, endometrial thickness, volume, echo, peristalsis, and blood flow.

##  Conflict of Interest

The authors declare that they have no conflict of interest to disclose. We uphold the principles of integrity and transparency in our research and have no personal or financial affiliations that could influence the outcomes or interpretation of our work.
